# Maternal complications within six weeks after primary caesarean section in Ghana: a prospective cohort study

**DOI:** 10.1186/s12884-026-09304-w

**Published:** 2026-06-03

**Authors:** Kwaku Asah-Opoku, Kofi Agyabeng, Joyce Browne, Mercy Nuamah, Nelson Damale, Rick Grobbee, Kitty Bloemenkamp, Marcus J Rijken

**Affiliations:** 1 Department of Obstetrics and Gynecology, University of Ghana Medical School, P.O. Box GP 4236, Accra, Ghana; 2 Department of Obstetrics and Gynaecology, Korle-Bu Teaching Hospital, Accra, Ghana; 3 Department of Obstetrics, Division Woman and Baby, WKZ Birthcentre, University Medical Centre, Utrecht University, Utrecht, The Netherlands; 4 Julius Centre for Health Sciences and Primary Care, Julius Global Health, University Medical Centre, Utrecht University, Utrecht, The Netherlands; 5 Department of Biostatistics, School of Public Health, University of Ghana, Accra, Ghana

**Keywords:** Caesarean section, Maternal, Complications, Six weeks, Ghana

## Abstract

**Background:**

Caesarean section (CS) rates are increasing globally. Though CSs are known to contribute to improving maternal and neonatal outcomes, they can result in short and long term complications. We sought to find out the maternal complications associated with primary CSs within six weeks post-delivery in a unique cohort.

**Methods:**

This was a prospective cohort study conducted at the Korle Bu Teaching hospital, Accra, Ghana. A total of 1013 women who had their primary CS procedure carefully documented between 1st January, 2018 to 14th January, 2020 were followed up to six weeks post -delivery and CS complications observed and documented. Adjusted Poisson Model with Robust Standard Error was used to determine incidence risk ratios for CS complications.

**Results:**

The primary CS maternal complication rate was 12.3% (CI-10.3-14.4). Surgical site infection (SSI) rate was 32/989(3.2%) and constituted 32/65(49.2%) of all the post-operative complications. Positive cultures for bacteria were obtained in 4/32(12.5%) of the women who had SSI. Factors associated with CS complications include: age 30–34 years compared to teenagers IRR 0.24 [CI: 0.08–0.73], being widowed IRR 7.09 [CI: 1.22–41.38], abnormal vaginal discharge during pregnancy IRR 2.13 [CI: 1.094.17] and electrocoagulation for dissection of subcutaneous tissue IRR 0.44 [CI: 0.21–0.91].

**Conclusion:**

Primary CS complication rate within the first 6 weeks post- partum is relatively low with the majority being surgical site infections. The risk factors associated with CS complications are; being a teenager, women who are widowed and recurrent abnormal vaginal discharge during pregnancy. The use of diathermy for CS is associated with less risk of CS complications. Identification of these factors and provision of appropriate support and treatment will help to reduce the complications associated with CSs at Ghana’s largest referral teaching hospital and enable women have a more positive pregnancy experience.

**Supplementary Information:**

The online version contains supplementary material available at 10.1186/s12884-026-09304-w.

## Background

The global Caesarean section (CS) rates have reached epidemic proportions [1, 2]. CS rates should not exceed 10–15% at population level [3] and maternal death rate does not decrease with CS rates above 19% [4]. Though CSs contribute to improving maternal and neonatal outcomes, they are associated with short and long term complications for the mother and neonate as well [5, 6]. Maternal morbidity from CS deliveries is twice that of vaginal delivery [7]. The maternal near- miss ratio associated with CSs is quoted as 36 per 1000 live births [8]. A quarter of all women who die in lower middle income countries are from CS [9]. In situations where there are no medical or obstetric indications for CS, the neonatal morbidities and mortalities are also worse compared to vaginal deliveries [10, 11]. Most of the short- term complications of CSs are recorded within 4 to 12 weeks post CS [12]. Short term maternal complications of CS include haemorrhage, surgical site infections, endometritis, wound haematomas, gaped wounds and post –operative pain [5, 6, 9], while respiratory distress syndrome and trauma are examples of complications in neonates [9, 13]. In most situations, CSs are conducted “too little, too late”, or “too many, too soon” [14]. Both circumstances can result in poor outcomes for the mother and neonate [15, 16]. A recent overview of systematic reviews on evidence based procedures for CS indicated the importance of relating surgical aspects of the procedure to specific outcomes such as complications [17]. These complications occur on a greater scale in low middle- income countries (LMICs) because of poor health systems comprising inadequate medical staff, poor access to medicines and drugs and poor health financing [6, 9]. Late reporting to health facilities and late referrals have also compounded the rate of post-CS complications in these arears [18]. These complications further stretch the already limited health facilities and staff in the LMICs [19]. In a recent systematic review and meta-analysis, the risk of maternal death from CS was 0.76% with Sub-Saharan Africa having the highest proportion and a fourth of all women who died in LMICs had undergone CS [9]. Most of the studies that have been published on maternal and fetal complications of CS have been carried out on CS in general without distinguishing between primary CSs and repeat CSs [9, 13, 20] while others have concentrated on complications associated with multiple CSs where the perceived risk is high [21, 22]. Most of the studies on complications of CS are retrospective in nature with the challenges such as lost data, incomplete data among others [22, 23]. Other studies on CS complications are cross-sectional [8]. A recent prospective study on complications of CS in Sierra Leone focused on perinatal outcomes [24]. The possibility of complications arising as a result of primary CSs are often downplayed. The documentation of these complications related to the primary CS is important as their occurrence increase the probability of complications in subsequent pregnancies.

The aim of this study was to determine the maternal complications of primary CS as well as the associated factors in our unique cohort and the complications related to the technical aspects of the operation.

## Methods

### Study design

This was a prospective cohort study conducted at the Korle Bu Teaching hospital, Accra, Ghana.

### Study setting

The study was carried out at the maternity unit of the Korle-Bu Teaching Hospital (KBTH), which is situated in Accra, the capital city of Ghana and serves as the main tertiary and referral centre for the Greater Accra and surrounding regions. KBTH is also the teaching hospital used for clinical teaching by the University of Ghana Medical School. Patients attending the antenatal clinic and gynaecology unit are of various socioeconomic, cultural and religious backgrounds. The total bed capacity of the obstetrics and gynaecology department is 375. The maternity unit has two functioning theatres. An average of 9294 deliveries are conducted yearly. Of these deliveries, 4332 are by CS giving a CS rate of 43.7%. Two thousand and thirteen of these CSs are electives and the rest are emergencies. 965 of these CSs are first time (primary) CSs. Two hundred and eighty one of these primary CSs are electives whiles the rest are emergencies. The vaginal birth after CS rate is 48.47% [25]. The CS are done under spinal anaesthesia unless there is a contraindication. All patients are given prophylactic Amoxicillin-Clavulanic acid and metronidazole intravenously.

### Study population

All women who underwent their primary CS at the Korle-Bu Teaching Hospital were included in the study.

#### Inclusion criteria

The study included women aged 18years and above who consented to the study and had their first CS.

#### Exclusion criteria

Unbooked clients.

Women who had peri-mortem CSs were excluded from the study.

### Sampling technique and sample size

#### Data collection procedure and study instrument

At the KBTH, thirteen different research assistants consisting of medical students on research internship and research assistants involved in research at the Department of Obstetrics and Gynaecology for more than 2 years were involved in the CS observations and data collection. All members of the team completed a research observation training and quality control checks. The senior investigators of this study were available for questions and clarifications throughout the study period.

Every included case was observed by the research assistants and documentations made using a standard case report form.

At the start of the study research assistants were trained in obtaining a visual analogue scale (VAS) score, how to conduct detailed CS procedure documentation, interview the women, and identify complications. After the 2 weeks research documentation training in the theater, they did 4 weeks double scoring with the principal investigator and data differences were clarified until there was congruence in terms of data collection results before they were left to collect data by themselves. Halfway through the study period a training was organized again to ensure that the observations and collection of data occurred as homogenously as possible.

Patients who were scheduled for elective CS had the study explained to them and a written informed consent obtained from them if they met the inclusion criteria. Women in labour also had the study explained to them and a written informed consent obtained that there will be observation and recording of their CS procedure if their delivery ended in CS. The research team run morning afternoon, evening and night shifts in the operating theatre rooms of the maternity block of the Department of Obstetrics and Gynaecology during which the CS conducted on all women who fit the inclusion criteria was observed. Patients were followed up until six weeks after the CS. For this study, a patient was adjudged to have a complication of CS if she had one of the following within 6 weeks post-partum: post-operative urinary tract infection, surgical site infection, subcutaneous haematoma, re- laparotomy, blood transfusion, length of stay beyond the routine schedule of days for admission, emergencies post-delivery requiring readmission post-operative ileus, post –operative ventilator support and post-partum haemorrhage, Surgical site infection was made using the CDC diagnostic criteria for surgical site infections but was adapted for the period of 6 weeks for the purposes of this study instead of 30 days [26] and maternal mortality from CS.

Data concerning sociodemographic characteristics, medical and obstetric history, medication use and antenatal care were retrieved from the patient’s folder and by interviewing the patients. Information on prescribed and administered analgesics immediately and 24 h-postoperatively, were retrieved from the patients’ treatment sheet and anesthetics records. Complications of CS as indicated in the checklist (Supplementary file 1) were noted.

#### Follow-up

After discharge, patients came for postnatal follow-up to the hospital at 1–2 weeks and then at 6 weeks. A member of the research team approached patients at the postnatal clinic to retrieve information collected by the doctors and interview the patient. If women did not attend the postnatal clinic, phone calls were made to retrieve information. At each follow up, complications of CS were noted. At 6 weeks, the principal investigator went through all the patients’ folders to double check and make sure that no complications had been missed.

#### Data management and analysis

The data collected were entered into KoBo Toolbox and extracted into Microsoft Excel file. The extracted data was then imported into STATA version 14 (Stata Corporation, College Station, TX, USA) for data management and analysis. Through continuous quality monitoring and record assessment, missing data was minimized as much as possible. All missing data were classified as missing completely at random. Descriptive statistics were presented as frequencies and percentages for categorical variables and mean with standard deviation for continuous variables. CS complication rate was calculated as the proportion of study sample with at least one CS complication within 6 weeks post-surgery. Surgical site infection (SSI) was defined using the CDC Criteria for SSI but modified to cover the period of 42 days instead of 30 days [26]. Chi-square test of association was used to test for association between background and Clinical characteristics and surgical activities (Determinant variables) and CS complication among women who had their first CS at the Korle-Bu Teaching Hospital (Outcome variable). Modified Poisson regression model with robust standard error was used to determine the effect of background and clinical characteristics and surgical activities on CS complication among women who had their first CS at the Korle-Bu Teaching Hospital. The effect size was reported as relative risk with their corresponding confidence intervals. Modified negative binomial regression model with robust standard error was used as sensitivity analysis. There was no data imputation for missing data. A complete case analysis of each variable was performed. All statistical test were done at 5% significance level. The study findings were reported on according to the STROBE guidelines for observational studies (Supplementary file 2).

#### Sample size determination

This manuscript is part of a larger study in which we aimed to associate the surgical technique to subsequent intra-abdominal adhesions. We referenced a retrospective analysis which showed that 62% of women with a previous CS had abdominal adhesions at the repeat CS [27]. In order to confirm this number in a prospective study, the sample size was calculated (Kish L ,1965) at 95% confidence interval with a 5% allowable margin of error and an assumption of a 30% loss to follow up. This resulted in a minimum sample size of 918. Patients are still being followed up for adhesion formation at the next CS.

## Results

**Fig. 1:**
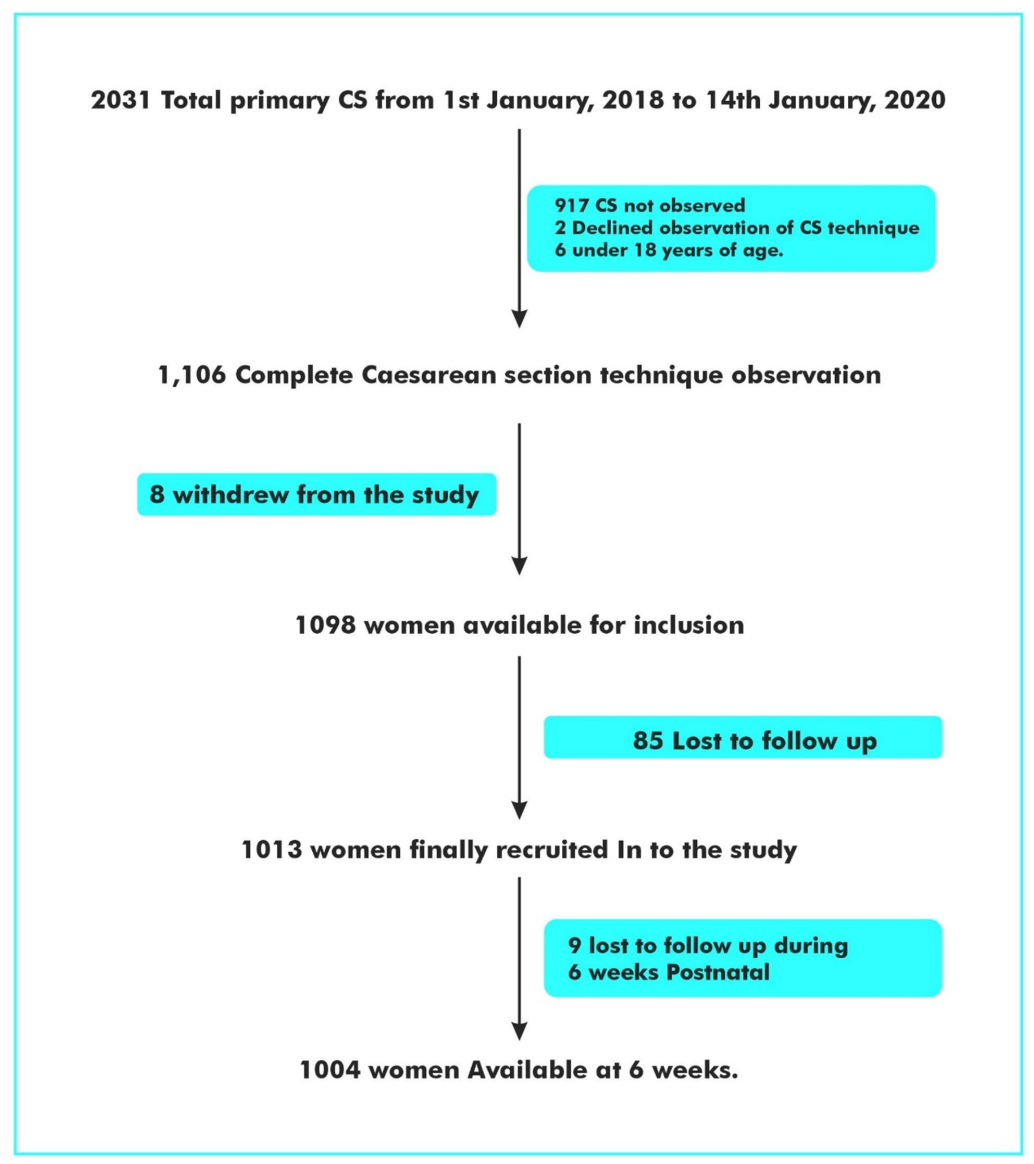
Flow chart of patient recruitment into the study

The recruitment process for participants in the study is shown in the flowchart (Fig. 1.)

The study involved 1004 women with a mean (SD) age of 30.3 ± 5.9years. About a quarter of the women had secondary (24.7%, 248/1004) or tertiary (25.9%, 260/1004) level of education. Most (75.4%, *n* = 757/1004) women were married and for 52.3% (*n* = 525/1004) this was their first pregnancy (Table 1).

**Table 1:** Sociodemographic characteristics of women who had their primary CS at the Korle-Bu Teaching Hospital between 1st January, 2018 to 14th January, 2020

Variable	Frequency	Percentage
Age (years)		
Mean ± SD	30.3 ± 5.9	
< 20	35	3.5
20–24	134	13.3
25–29	273	27.2
30–34	302	30.1
> 34	252	25.1
Missing	8	0.8
Highest level of education		
No formal education	53	5.3
Primary	74	7.4
Junior Secondary School	361	36.0
Senior Secondary School	248	24.7
Tertiary	260	25.9
Missing	8	0.8
Occupation		
Unemployed	95	9.5
Student	31	3.1
Trader/Farmer	368	36.7
Salaried worker (public)	97	9.7
Salaried worker (Private)	125	12.5
Other	280	27.9
Missing	8	0.8
Marital Status		
Married	757	75.4
Single	170	16.9
Cohabiting/engaged	63	6.3
Widowed	4	0.4
Missing	10	1
Parity		
0	525	52.3
1	176	17.5
2–4	272	27.1
>4	31	3.1
Gravidity		
1	293	29.2
2–3	405	40.3
4–6	258	25.7
>=7	47	4.7
Missing	1	0.1
BMI		
Normal	90	9
Overweight/Obese (BMI > 25)	652	64.9
Missing	262	26.1

The mean (SD) gestational age at delivery was 38.4 ± 3.0 weeks and 20.1% (*n* = 202/1004) had a preterm birth. More than 80% (*n* = 817/1004) of the CS were emergency procedures and most of the surgeons (67.4%, 677/1004) were senior residents. Almost all the women received Spinal anaesthesia (92.1%, *n* = 925/1004), were shaved on the ward (94.5%, *n* = 949/1004), disinfected with Chlorhexidine followed by alcohol (94.0%, *n* = 944/1004), and had a transverse skin incision (98.0%, *n* = 984/1004) (Table 2) (manuscript submitted).

**Table 2:** Clinical characteristics and surgically related activities for women who had their primary CS at the Korle-Bu Teaching Hospital between 1st January, 2018 to 14th January, 2020

Variable	Frequency	Percentage
Gestational age at delivery		
Mean ± SD (weeks)	38.4 ± 3.0	
Term	799	79.6
Preterm	202	20.1
Missing	3	0.3
Type of CS		
Elective	171	17
Emergency	817	81.4
Missing	16	1.6
Level of surgeon		
Consultant/Senior Specialist	73	7.3
Senior resident	677	67.4
Junior resident	192	19.1
Medical/House officer	35	3.5
Missing	27	2.7
Type of anesthesia		
Epidural	10	1
Spinal	925	92.1
General	57	5.7
Missing	12	1.2
Shaving before surgery		
No	37	3.7
Yes: ward	949	94.5
Yes: Theater	18	1.8
Disinfection of skin at surgery		
Chlorhexidine alcohol mixture	57	5.7
Chlorhexidine followed by alcohol	944	94.0
Missing	3	0.3
Type of skin incision		
Transverse	984	98.0
Vertical	13	1.3
Missing	7	0.7
Skin incision		
Cutting skin and subcuticular tissues together	291	29
Cutting skin and subcuticular tissues separately	698	69.5
Missing	15	1.5
Removal of placenta		
Controlled cord traction	913	90.9
Manual	79	7.9
Missing	12	1.2
Peritoneal closure		
Closed	378	37.6
Non-Closure	607	60.5
Missing	19	1.9
Subcutaneous suturing		
Yes	668	66.5
No	311	31
Missing	25	2.5
Bupivacaine skin infiltration		
Yes	69	6.9
No	883	87.9
Missing	52	5.2
Abnormal vaginal discharge		
Yes	61	6.1
No	925	92.1
Missing	18	1.8
Level of post-operative pain		
No Pain	6	0.6
Mild	279	27.8
Moderate	494	49.2
Severe	207	20.6
Missing	18	1.8

The complication rate was 12.3% (CI-10.3-14.4). Surgical site infection (SSI) rate was 32/989 = 3.2% and constituted 32/65(49.2%) of all post-operative complications. Positive cultures for bacteria were obtained in 4/32(12.5%) of the women who had SSI. E. coli and proteus mirabilis were cultured in 2 of the patients and Klebsiella pneumonia in another 2. Table 3; shows the complications of the primary CS up to 6 weeks post-partum.

**Table 3:** Complications up to 6 weeks post first CS

Complications	Frequency	Percentage
Postoperative urinary tract infection (*n* = 987)	10	1.0%
Surgical site infection (*n* = 989)	32	3.2%
Postoperative subcutaneous haematoma (*n* = 990)	2	0.2%
Postoperative ileus (*n* = 991)	1	0.1%
Postoperative ventilator support(*n* = 987)	4	0.4%
Obstetric haemorrhage (*n* = 987)	16	1.6%
Blood transfusion (*n* = 987)	61	6.2%

Differences in n are due to missing data

There was one maternal death. This woman had a CS because of eclampsia. She developed multiple organ failure from the severe eclampsia a week after the CS. Post-operative ventilator support was needed in 4/987(0.4%) women:, one had a cardiac-arrest at the post CS recovery unit and had spontaneous return of cardiac activity after cardiopulmonary resuscitation, one had severe anaemia post Caesarean hysterectomy for postpartum haemorrhage from uterine atony and had poor respiratory effort post- surgery, the third woman had sickle cell disease with acute chest syndrome and the fourth woman admitted to the Intensive Care Unit (ICU) was for severe sepsis with respiratory distress post CS. All these four women survived.

Association between patient characteristics/surgical procedures and 6 weeks post- CS complications.

From the Chi-square test of association, no statistically significant association was found between patient characteristics, surgical techniques and post CS complications up to 6 weeks post-partum. This is shown in Table 4.

**Table 4:** Association between patient characteristics/surgical procedures and 6 weeks post- CS complications

Factor	Complication		*p*-value
N	No	Yes	
Age			0.067
< 20	26 (74.3)	9 (25.7)	
20–24	116 (86.6)	18 (13.4)	
25–29	237 (86.8)	36 (13.2)	
30–34	274 (90.7)	28 (9.3)	
> 34	220 (87.3)	32 (12.7)	
Gestational age at delivery			0.38
Term	698 (87.4)	101 (12.6)	
Preterm	181 (89.6)	21 (10.4)	
Highest level of education			0.27
No formal education	48 (90.6)	5 (9.4)	
Primary	66 (89.2)	8 (10.8)	
Junior Secondary School	307 (85)	54 (15)	
Senior Secondary School	217 (87.5)	31 (12.5)	
Tertiary	236 (90.8)	24 (9.2)	
Occupation			0.42
Unemployed	84 (88.4)	11 (11.6)	
Student	25 (80.6)	6 (19.4)	
Trader/Farmer	314 (85.3)	54 (14.7)	
Salaried worker (public)	89 (91.8)	8 (8.2)	
Salaried worker (Private)	113 (90.4)	12 (9.6)	
Other	249 (88.9)	31 (11.1)	
Marital Status			0.49
Married	668 (88.2)	89 (11.8)	
Single	148 (87.1)	22 (12.9)	
Cohabiting/engaged	52 (82.5)	11 (17.5)	
Widowed	3 (75)	1 (25)	
Abnormal vaginal discharge			0.15
Yes	50 (82)	11 (18)	
No	816 (88.2)	109 (11.8)	
Level of post-operative pain			0.13
No Pain	5 (83.3)	1 (16.7)	
Mild	253 (90.7)	26 (9.3)	
Moderate	432 (87.4)	62 (12.6)	
Severe	173 (83.6)	34 (16.4)	
Level of Surgeon			0.19
Consultant/Senior specialist	65 (89)	8 (11)	
Senior resident	599 (88.5)	78 (11.5)	
Junior resident	159 (82.8)	33 (17.2)	
Medical/House officer	33 (94.3)	2 (5.7)	
Parity			0.62
0	463 (88.2)	62 (11.8)	
1	156 (88.6)	20 (11.4)	
2–4	237 (87.1)	35 (12.9)	
> 4	25 (80.6)	6 (19.4)	
Type of CS			0.20
Elective	155 (90.6)	16 (9.4)	
Emergency	712 (87.1)	105 (12.9)	
Type of anesthesia			0.33
Combined spinal/ Epidural	8 (80)	2 (20)	
Spinal	816 (88.2)	109 (11.8)	
General	47 (82.5)	10 (17.5)	
Shaving before surgery			0.19
No	30 (81.1)	7 (18.9)	
Yes: ward	837 (88.2)	112 (11.8)	
Yes: Theatre	14 (77.8)	4 (22.2)	
Disinfection of skin at surgery			0.21
Chlorhexidine alcohol mixture	53 (93)	4 (7)	
Chlorhexidine followed by alcohol	825 (87.4)	119 (12.6)	
Type of skin incision			0.72
Transverse	865 (87.9)	119 (12.1)	
Vertical	11 (84.6)	2 (15.4)	
Skin incision			0.076
Cutting skin and subcuticular tissues together	264 (90.7)	27 (9.3)	
Cutting skin and subcuticular tissues separately	605 (86.7)	93 (13.3)	
Placental separation			0.059
Controlled cord traction	806 (88.3)	107 (11.7)	
Manual	64 (81)	15 (19)	
Peritoneal closure			0.70
Closure	335 (88.6)	43 (11.4)	
Non-Closure	533 (87.8)	74 (12.2)	
Subcutaneous suturing			0.55
Yes, describe	589 (88.2)	79 (11.8)	
No	270 (86.8)	41 (13.2)	
Bupivacaine for skin infiltration post CS			0.33
Yes	63 (91.3)	6 (8.7)	
No	771 (87.3)	112 (12.7)	
BMI			0.48
Normal	81 (90)	9 (10)	
Overweight/Obese	570 (87.4)	82 (12.6)	

After adjusting for various confounders, teenagers had the highest risk for developing complications, while women aged 30–34 years had a 76% reduced risk within 6 weeks post CS compared to other age groups[RR 0.24, 95%CI: 0.08–0.73]. Women who had abnormal vaginal discharge during pregnancy had a two- fold increase in the risk for a CS complication [[RR 2.13, 95%CI: 1.09–4.17]. Women who were widowed during the postpartum period had a 7 fold risk for developing complications [RR 7.09, 95%CI: 1.22–41.38]. The use of electrocoagulation for dissection of subcutaneous tissue was associated with a 56% decrease in the risk for CS complication compared to other methods of subcutaneous tissue dissection. [RR 0.44, 95%CI: 0.21–0.91]. There were no other significant risk factors for developing CS Complications at 6weeks postpartum. The risk factors for developing post CS complications are illustrated in Table 5.

**Table 5:** Multiple logistic regression for association between patient characteristics/surgical procedures and 6 weeks post- CS complications

	Adjusted Poisson Model with Robust Standard Error		Adjusted Negative Binomial Model with Robust Standard Error	
	RR [95% CI]	*P*-value	RR [95% CI]	*P*-value
Age				
< 20	1		1	
20–24	0.47 [0.2–1.11]	0.084	0.47 [0.2–1.11]	0.084
25–29	0.54 [0.22–1.29]	0.164	0.54 [0.22–1.29]	0.164
30–34	**0.24 [0.08–0.73]**	**0.012**	**0.24 [0.08–0.73]**	**0.012**
> 34	0.34 [0.11–1.08]	0.067	0.34 [0.11–1.08]	0.067
Gestational age at delivery				
Term	1		1	
Preterm	0.69 [0.32–1.47]	0.332	0.69 [0.32–1.47]	0.332
Highest level of education				
No formal education	1		1	
Primary	1.51 [0.37–6.09]	0.562	1.51 [0.37–6.09]	0.562
Junior Secondary School	2.35 [0.8–6.94]	0.121	2.35 [0.8–6.94]	0.121
Senior Secondary School	1.91 [0.63–5.81]	0.254	1.91 [0.63–5.81]	0.254
Tertiary	2.25 [0.57–8.89]	0.248	2.25 [0.57–8.89]	0.248
Occupation				
Unemployed	1		1	
Student	1.13 [0.31–4.08]	0.857	1.13 [0.31–4.08]	0.857
Trader/Farmer	1.94 [0.9–4.2]	0.09	1.94 [0.9–4.2]	0.09
Salaried worker (public)	0.58 [0.13–2.68]	0.488	0.58 [0.13–2.68]	0.488
Salaried worker (Private)	0.65 [0.19–2.29]	0.508	0.65 [0.19–2.29]	0.508
Other	0.77 [0.32–1.88]	0.573	0.77 [0.32–1.88]	0.573
Marital Status				
Married	1		1	
Single	0.93 [0.48–1.77]	0.815	0.93 [0.48–1.77]	0.815
Cohabiting/engaged	1.86 [0.9–3.87]	0.095	1.86 [0.9–3.87]	0.095
Widowed	**7.09 [1.22–41.38]**	**0.029**	**7.09 [1.22–41.38]**	**0.029**
Abnormal vaginal discharge				
Yes	**2.13 [1.09–4.17]**	**0.026**	**2.13 [1.09–4.17]**	**0.026**
No	1		1	
Level of post-operative pain				
No Pain	1		1	
Mild	0.39 [0.1–1.52]	0.175	0.39 [0.1–1.52]	0.175
Moderate	0.44 [0.11–1.69]	0.23	0.44 [0.11–1.69]	0.23
Severe	0.83 [0.23–3.09]	0.787	0.83 [0.23–3.09]	0.787
Level of Surgeon				
Consultant/Senior specialist	1		1	
Senior resident	1.12 [0.43–2.9]	0.822	1.12 [0.43–2.9]	0.822
Junior resident	1.72 [0.62–4.73]	0.294	1.72 [0.62–4.73]	0.294
Medical/House officer	0.37 [0.04–3.2]	0.366	0.37 [0.04–3.2]	0.366
Parity				
0	1		1	
1	0.97 [0.48–1.97]	0.937	0.97 [0.48–1.97]	0.937
2–4	1.03 [0.45–2.38]	0.938	1.03 [0.45–2.38]	0.938
> 4	1.46 [0.36–5.97]	0.601	1.46 [0.36–5.97]	0.601
Type of CS				
Elective	1		1	
Emergency	1.54 [0.85–2.77]	0.154	1.54 [0.85–2.77]	0.154
Type of anesthesia				
General	1		1	
Spinal	0.23 [0.27–1.38]	0.237	0.23 [0.27–1.38]	0.237
Combined Spinal/Epidural	3.67 [0.68–19.80]	0.130	3.67 [0.68–19.80]	0.130
Shaving of surgical site				
No	1		1	
Yes: ward	0.81 [0.36–1.82]	0.609	0.81 [0.36–1.82]	0.609
Yes: Theater	1.14 [0.23–5.59]	0.875	1.14 [0.23–5.59]	0.875
Disinfection of skin at surgery				
Chlorhexidine alcohol mixture	1		1	
Chlorhexidine followed by alcohol	2.16 [0.57–8.25]	0.259	2.16 [0.57–8.25]	0.259
Type of skin incision				
Transverse	1		1	
Vertical	2.84 [0.67–12.06]	0.157	2.84 [0.67–12.06]	0.157
Skin incision				
Cutting skin and subcuticular tissues together	1		1	
Cutting skin and subcuticular tissues separately	1.1 [0.65–1.86]	0.72	1.1 [0.65–1.86]	0.72
Dissection of subcutaneous tissues				
Scalpel	1		1	
Scissor	0.74 [0.18–3.15]	0.688	0.74 [0.18–3.15]	0.688
Electrocoagulation	**0.44 [0.21–0.91]**	**0.028**	**0.44 [0.21–0.91]**	**0.028**
Blunt	0.64 [0.38–1.08]	0.097	0.64 [0.38–1.08]	0.097
Placental separation				
Controlled cord traction	1		1	
Manual	1.38 [0.76–2.52]	0.29	1.38 [0.76–2.52]	0.29
Peritoneal closure				
Closure	1		1	
Non-Closure	0.84 [0.51–1.37]	0.482	0.84 [0.51–1.37]	0.482
Subcutaneous suturing				
Yes	0.82 [0.52–1.29]	0.385	0.82 [0.52–1.29]	0.385
No	1		1	
Bupivacaine for skin infiltration post CS				
Yes	0.41 [0.12–1.34]	0.14	0.41 [0.12–1.34]	0.14
No	1		1	
BMI				
Normal	1		1	
Overweight/Obesity	1.74 [0.8–3.78]	0.163	1.74 [0.8–3.78]	0.163

*RR* Relative risk, *BMI* Body Mass Index, *CI* Confidence Interval, *CS *Caesarean section

Bold face figures-Statistically significant

## Discussions

This is the first study in Africa that has outlined the complications of the primary CS in a large cohort of women who had their CS technical procedures precisely documented. The CS complication rate in the cohort was 12.3% with majority of the complications (49.2%) being surgical site infections. Bacterial swab cultures were positive in 12.5% of the surgical infections. 6.2% of the women after their primary CS received blood transfusion. Post- partum mothers aged 30–34 years compared to teenagers and women in whom electrocoagulation was used for dissection of the subcutaneous tissue had the lowest risk of complications. However, women who had abnormal vaginal discharge during pregnancy and women who were widowed during the postpartum period were at increased risk. There were no other significant risk factors for developing CS complications at 6weeks postpartum. The single case of maternal mortality recorded in this cohort of women was from complications of eclampsia and not the CS.

The complication rate obtained in this study is less than the 17.1% complication rate obtained seven days post CS in a prospective study in 22 African countries [28]. Even though this current study had a follow-up to six weeks post- delivery and therefore expected to pick a greater proportion of complications, the prospective study in Africa included all CS, whereas the current study was limited to first CS only. Deliveries from repeat CS are known to result in greater complications [5, 29]. 

The surgical site infection rate in this study was far less than that obtained from a previous study in the same setting in 2017, where repeat CS were included as well [30]. Another reason for the relatively low rate of complications is the experience of the surgical team as described in a study from Canada [31]. Indeed, the majority of CS were carried out by specialist and consultant obstetrician gynaecologists in KBTH compared to the situation in other levels of care where less experienced staff perform CS.

The SSI rate in this study was also a far less than that recorded from a prospective study done in Tanzania which recorded a SSI rate of 48% [32]. However, in this latter study, repeat CS were included and also only 2.1% of the women received pre-incision antibiotics, whereas in our study all women received prophylaxis with Amoxicillin-Clavulanic acid and Metronidazole given intravenously.

Surgical site infections were the main contributor to the CS complications at the Korle-Bu Teaching Hospital in Accra, Ghana. This finding is contrary to the already mentioned large prospective study of Bishop et al. [28] where the leading post-CS complication was haemorrhage.

Haemorrhage was prevalent in this latter study due to the fact that the multinational study included repeat CSs which are more likely to be associated with haemorrhage compared to primary CS.

Despite the fact that obstetric haemorrhage occurred in 1.6% of the women in this cohort, blood transfusion was carried out in 6.2% of them. Aside the 16 women with PPH that were transfused, the other cases had anaemia prior to surgery and therefore even with normal blood losses at surgery required blood transfusion during admission. This is less than the 20.8% of women who received blood transfusion among patients who had CS at the Lagos State University Hospital in Nigeria [33], where both primary and repeat CS were included. Previous surgeries are associated with increased incidence of adhesions and placental abnormalities such as placenta accreta syndrome which account for increased blood loss that necessitated blood transfusion [34]. 

In this study, teenagers were more likely to develop CS complications compared to those aged 30 to 34 years. This agrees with the findings of other studies in which teenagers have been found to have higher rates of obstetric complications such as puerperal sepsis, urinary tract infection and maternal death [35, 36]. Poor nutrition, low level of education, poor social supports, and poor antenatal care are some of the factors accounting for obstetric complications among teenagers [37, 38]. 

Post-partum women who were widowed had a seven fold risk for CS complications compared to married women. Being socially disadvantaged has been associated with stress, poor antenatal engagement and increased risks of obstetric outcomes such as postpartum haemorrhage [39]. This stresses the importance of special social support for widows in the post- partum period and should be highlighted in studies and clinical care.

Women with recurrent abnormal vaginal discharge whiles pregnant had twice the risk for CS complications compared to those who did not have. The commonest cause of abnormal vaginal discharge is bacterial vaginosis [40]. Abnormal vaginal discharge reported by these women while they were pregnant, maybe as a result of bacterial vaginosis with consequent complications such as endometritis and other surgical site infections. Bacterial vaginosis has been shown to lead to intrapartum chorioamnionitis and subsequent development of postpartum endometritis [40]. This is a modifiable risk and therefore calls for regular investigation and complete treatment of abnormal vaginal discharges that occur during pregnancy.

In this study, use of electrocoagulation for dissecting subcutaneous tissue was associated with a 56% decrease in the risk of complications of CS compared to the use of the scalpel. The finding of this study is at variance with that of Gupta et al. in India [41] who found wound complication rates to be similar between the use of electrocautery and scalpel and also that of Charoenkwan et al. in a Cochrane review [42]. This Cochrane review however included other surgeries in addition to CSs as well as repeat Caesarean surgeries. The lower complication rate of CSs when diathermy is used as observed in this study could be attributed to reduced risk of bleeding associated with the use of the diathermy and reduction in time duration of surgery. Electrocautery for dissection of the subcutaneous tissue is also more likely to reduce the risk of wound haematomas compared to the use of scalpel as it coagulates vessels and reduces bleeding. A recent review of systematic reviews published in the Lancet has called for more high quality systematic reviews that will compare individual methods of abdominal entry procedures [17]. 

From this study, there was a single mortality from multiple organ failure from eclampsia which was unrelated to CS and also the four cases that were put on a ventilator at the ICU and survived portrays specialized care by the team of attending physicians, surgeons and anaesthesiologists. This finding is in sharp contrast to that of a systematic review by Sohby et al. in low and middle income countries which showed the risk of maternal death in women who had a caesarean section to be 7·6 per 1000 procedures [9]. The study by Sobhy et al. however did include repeat CS and also settings with lower level of care compared to our current study.

The strength of this study is that it is one of the first large studies in Africa that has examined the maternal complications of precisely documented first CS procedures within the first six weeks postpartum. The prospective nature of the study also allowed for estimation of risk ratios. The limitation of the study is that a total of 13 research assistants were used for the surgical technique observations. This could have introduced some heterogeneity in the observations made and also some surgical technique steps could have been misobserved especially in situations where the surgeons were very quick with their techniques. This limitation was however ameliorated by the fact that all members of the research team completed a research observation training which was repeated midway through the study. There were also quality checks on the data collected and the surgeons who conducted the CS were always available for clarification of the steps they used in conducting the CS. Each participant was interviewed before discharge from the hospital and at one week and six weeks post CS. This enabled us to trace all cases of wound infection to the best of our abilities. Another limitation of this study is that blood loss estimates were done by visual estimation of blood loss in the suction bottle, operating field and soaked swabs.

## Conclusion

CS complication rate within the first 6 weeks post- partum for primary CSs are relatively low with the majority being surgical site infections. While women who were widowed, and women with recurrent abnormal vaginal discharge during pregnancy had the most post CS complications, women aged between 30 and 34 years compared to teenagers and when diathermy for CS was used had the least complications. Identification of these factors and provision of appropriate support and treatment will help to reduce the complications associated with CSs at Ghana’s largest referral teaching hospital and enable women have a more positive pregnancy experience.

## Supplementary Information

Supplementary Material 1

Supplementary Material 2

## Data Availability

All data supporting the findings of this study are available upon reasonable request.

## Abbreviations

CDC: Centers for Disease Control and Prevention
CS: CS
IRR: Incidence Risk Ratio
KBTH: Korle-Bu Teaching Hospital
LMIC: Lower-Middle-Income Country
STROBE: STrengthening the Reporting of OBservational Studies in Epidemiology
VAS: Visual Assessment Score
WHO: World Health Organization
