# Supplementary material for: Maternal complications within six weeks after primary caesarean section in Ghana: a prospective cohort study
Source: BMC Pregnancy Childbirth. 2026 Jun 3;26:866. doi: 10.1186/s12884-026-09304-w (PMC13455310; doi:10.1186/s12884-026-09304-w)
Supplement: Supplementary file 1 [file 12884_2026_9304_MOESM1_ESM.docx]

Supplementary file 1

**QUESTIONNAIRE CS COMPLICATION STUDY – CASE REPORT FORM (CRF)**

| 1. Weight at 20 weeks | \|__\|__\|__\|.\|__\| Kg |  |
| --- | --- | --- |
| 2. Last documented weight before delivery | \|__\|__\|__\|.\|__\| Kg  Gestational age: | Weeks |
| 3. Height | \|__\|.\|__\|__\| M |  |
| 4. Time of admission on the labor ward | \|__\|__\|:\|__\|__\| | |
| 5. Fetal weight | \|__\|__\|__\|.\|__\| g |  |
| 6. Admission to NICU | [0]□ Yes | [1]□ No |
| 7. If yes for question 6: reason |  | |
| 8. Fetal resuscitation | [0]□ Yes | [1]□ No |
| 9. Postoperative pain assessment (direct) | 0 1 2 3 4 5 6 7 8 9 10 | |
| 10. Type of post-operative analgesic | [0]□ IM Pethidine  [1]□ Suppository Paracetamol  [2]□ Suppository Diclofenac | [3]□ IV Paracetamol  [4]□ Other: |
| 11. Postoperative pain assessment (24h) | 0 1 2 3 4 5 6 7 8 9 10 | |
| 12. Type of post-operative analgesic | [0]□ IM Pethidine  [1]□ Suppository Paracetamol  [2]□ Suppository Diclofenac | [3]□ IV Paracetamol  [4]□ Other: |
| 13. Postoperative urinary tract infection | [0]□ Yes | [1]□ No |
| 14. If yes for question 13: urine collection for culture and sensitivity performed | [0]□ Yes | [1]□ No |
| 15. If yes for question 14: what organism was cultured |  | |
| 16. Postoperative wound infection | [0]□ Yes | [1]□ No |
| 17. If yes for question 16: wound swab taken | [0]□ Yes | [1]□ No |
| 18. If yes for question 17: what organism was cultured |  | |
| 19. Postoperative subcutaneous hematoma | [0]□ Yes | [1]□ No |
| 20. Emergencies developed post-surgery | [0]□ Yes | [1]□ No |
| 21. If yes for question 20: what emergencies |  | |
| 22. If yes for question 20: what interventions were carried out | [0]□ Use of uterotonics  [1]□ Condom catheter | [2]□ Relaparotomy  [3]□ Other: |
| 23. If relaparotomy: what specific procedure | [0]□ Compression sutures  [1]□ Suturing of bleeding vessels | [2]□ Hysterectomy  [3]□ Other: |
| 24. Blood transfusion | [0]□ Yes | [1]□ No |
| 25. If yes for question 24: how many units of blood were transfused? |  | Units |
| 25. Length of hospital stay |  | days |
